# Differential effects of ABTS-assisted laccase pretreatment on solidification and dewatering of humic-rich soil

**DOI:** 10.3389/fbioe.2026.1996007

**Published:** 2026-10-19

**Authors:** Xuanyu Chen, Prithvendra Singh, Kaiwei Luo, Qianwei Ma, Ningjun Jiang

**Affiliations:** 1 Institute of Geotechnical Engineering, Southeast University, Nanjing, Jiangsu, China; 2 Jiangsu Key Laboratory of Low Carbon and Sustainable Geotechnical Engineering, Nanjing, Jiangsu, China; 3 Department of Civil Engineering, Indian Institute of Technology Bombay, Mumbai, Maharashtra, India

**Keywords:** composite-binder stabilization, enzymatic pretreatment, geotextile-tube, humic-rich soil, laccase, redox mediator, slurry dewatering

## Abstract

**Introduction:**

Humic organic matter interferes with mineral-binder reactions and solid-liquid separation, limiting stabilization and dewatering of organic-rich soils and slurries.

**Methods:**

This study investigated laccase (LAC) pretreatment of an artificial humic-rich soil containing 10% humic acid, with 2,2’-azino-bis(3-ethylbenzothiazoline-6-sulfonic acid) (ABTS) as a redox mediator, and compared its effects on composite-binder solidification and slurry dewatering. Solidification used 8% composite curing agent (CSC; carbide residue:gypsum:cement = 20:1:1) after 24 h pretreatment.

**Results:**

At 20,000 and 40,000 U kg-1 dry soil, 14-d unconfined compressive strength (UCS) reached 841.4 and 818.3 kPa, respectively, versus 434.9 kPa for the control; 20,000 U kg-1 was therefore selected. In the component-comparison test, 14-d UCS was 797.4 kPa with LAC, 630.6 kPa with LAC+ABTS, 518.0 kPa with LAC+ABTS+CMCS, and 410.6 kPa for the control. In contrast, mediator addition benefited slurry treatment: LAC+ABTS followed by polyaluminum chloride (PAC) and carboxymethyl chitosan (CMCS) reduced supernatant and vacuum-filtrate turbidity and cake water content relative to LAC+PAC+CMCS. In the geotextile-tube test, LAC+ABTS+PAC+CMCS gave the greatest cumulative drainage (1702.8 mL) and the lowest central-region cake water content (84.94%). Dissolved organic carbon (DOC) decreased further after ABTS addition even though UCS declined, whereas LAC alone produced the densest interparticle morphology and the greatest local Ca enrichment without altering the dominant crystalline-phase assemblage.

**Discussion:**

These results show that laccase-based pretreatment should be formulated according to the intended engineering process.

## 1 Introduction

Excavation, tunnelling, dredging, and underground construction generate large volumes of fine-grained soil and high-water-content slurry that must be dewatered, stabilized, or both before transport, reuse, and disposal. When the excavated material is rich in humic substances, the central difficulty is not simply low intrinsic strength or excess water, but persistent organic interference at reactive interfaces. Carboxyl, phenolic, and other oxygen-containing groups in humic matter adsorb on mineral and binder surfaces, complex Ca- and Al-bearing species, retain water, and alter particle associations. In compacted binder-treated soils, these effects restrict particle-binder contact, suppress hydration-product development, and weaken the continuity of the load-bearing cemented network. Recent studies on humic-acid-bearing cemented soils and dredged sludge have consistently linked increasing organic content with strength loss, coarser pore structures, and inhibited formation of cementitious products, while oxidation-assisted treatment has shown that disrupting the organic barrier can restore part of the stabilization capacity (Cao et al., 2022; Huang et al., 2023; Yuan et al., 2025; Yu et al., 2025). Increasing cementitious reagent dosage can partly compensate for this inhibition, but it does not remove the interfacial cause of the problem. Waste-derived Ca-rich binders based on calcium carbide residue offer a lower-resource option for soil improvement and by-product valorization (Yu et al., 2024; Julphunthong et al., 2024; Moazami et al., 2025), yet their performance in humic-rich matrices remains constrained when the organic layer continues to occupy the reaction interface. In slurry, humic-rich fine particles remain highly hydrated and colloidally stable and can form compressible cakes with poor drainage continuity. Pretreatment should therefore modify the humic-rich interface before binder addition or coagulation/flocculation rather than relying solely on higher reagent dosages.

Biological reactions offer a means of modifying these interfaces before conventional geotechnical treatment. Bio-mediated geotechnics has already established that biochemical processes can be translated into controllable changes in mineral growth, interparticle cementation, stiffness and strength (Liu et al., 2018; Liu et al., 2019). Related studies have further extended this framework to liquefaction response and microscale crystal-growth processes (Xiao et al., 2018; Xiao et al., 2021; Xiao et al., 2022). More recent studies have treated soil biocementation explicitly as a coupled bioengineering process in which biological activity, transport, reaction distribution, and mineral precipitation jointly determine engineering performance (Chen et al., 2023; Maureira et al., 2024; Li et al., 2024). Most of this work, however, relies on biomineralization to create a new cementing phase. Much less attention has been paid to using extracellular enzymes to modify existing organic inhibitors before conventional solidification or dewatering. Laccase is well suited to this role because it oxidizes a broad range of phenolic and aromatic substrates under mild aqueous conditions using molecular oxygen as the terminal electron acceptor (Vaithyanathan et al., 2022; Dong et al., 2023; Abdi Dezfouli and Esmaeilidezfouli, 2024). Its effective reaction domain can be extended with a low-molecular-mass redox mediator such as 2,2′-azino-bis (3-ethylbenzothiazoline-6-sulfonic acid) (ABTS), which transfers oxidative equivalents from the enzyme to less accessible substrates (Gu et al., 2021; Wang et al., 2024; Aghaee et al., 2024). For humic-rich soil, however, a larger reaction domain cannot be assumed to be universally beneficial. Humic substances themselves participate in laccase-driven transformations and can also aggregate with or inhibit laccase, so enzyme performance depends on the physicochemical environment in which the catalytic system operates (Lopes et al., 2025; Park et al., 2021). The engineering question is therefore not whether ABTS increases laccase reactivity in isolation, but whether the resulting spatial pattern of organic transformation is favorable for the subsequent engineering process.

That distinction becomes critical because compacted solidification and slurry dewatering impose different structural requirements on the same material. Laccase-driven oxidation of phenolic constituents can promote cleavage, coupling, humification, and redistribution among extractable and non-extractable organic fractions rather than a single pathway of complete organic removal (Li et al., 2022; Zhou et al., 2022; Qiao et al., 2024). During solidification, the mechanically important locations are the particle-binder contacts where hydration products and Ca-rich precipitates must develop into continuous bridges. Localized modification of the organic coating at these contacts can improve cementation even when the overall change in organic matter is limited. In a high-water slurry, by contrast, performance is governed by suspension destabilization, polymer adsorption and bridging, floc size and strength, bound-water release, cake compressibility, and the persistence of connected drainage pores. Recent dewatering studies show that zeta-potential reduction alone cannot predict filtration behavior (Wu et al., 2021; Liu et al., 2024). After charge neutralization, floc architecture, bound-water distribution, cake compressibility, and pore structure continue to control dewaterability and filtration resistance (Tan et al., 2024; Wang H.-F. et al., 2025; Zhang Z. et al., 2024). The difference becomes larger when bench filtration is transferred to a geotextile tube. Flocculated dredged slurries in geotextile systems undergo progressive particle retention and cake development during filtration (Lu et al., 2024; Wang T. et al., 2025; Zhang H. et al., 2024). As the retained layer thickens, consolidation and evolving hydraulic resistance become increasingly important, so rapid initial water release does not necessarily translate into the greatest final drainage or the lowest cake water content (Bulut and Sengul, 2025; Karadoğan et al., 2022; Zhang et al., 2022). It remains unclear whether the same enzymatic pretreatment should use the same composition for compacted solidification and slurry dewatering. Without this comparison, mediator selection may be based on biochemical activity alone rather than on the engineering response of interest.

This study used an artificial humic-rich soil containing 10% humic acid to compare laccase pretreatment in CSC solidification and slurry dewatering. For solidification, the treated soil was mixed with 8% of a self-developed calcium-rich composite curing agent (CSC; carbide residue:gypsum:cement = 20:1:1 by mass). For dewatering, the same OH solids were dispersed as a high-water-content slurry and conditioned with polyaluminum chloride (PAC) followed by carboxymethyl chitosan (CMCS) or cationic polyacrylamide (cPAM). The experimental program comprised four parts: a laccase-dose comparison for CSC solidification, a component-comparison test of LAC, LAC + ABTS, and LAC + ABTS + CMCS, bench-scale coagulation/flocculation and vacuum filtration, and geotextile-tube dewatering. Given the different structural requirements of the two processes, this study tested whether ABTS-assisted laccase would affect compacted solidification and slurry dewatering differently. Dissolved organic carbon (DOC) analysis and ultraviolet–visible (UV–Vis) spectroscopy were used to track changes in the extractable and chromophoric organic fractions, for which optical indices provide established operational descriptors of dissolved organic matter composition and transformation (D’Andrilli et al., 2022; Korak and McKay, 2024; Begum et al., 2023); SEM, EDS, XRD, and FTIR were used to relate these changes to the solidified matrix and conditioned cakes. Accordingly, this study examines laccase pretreatment as a biochemical conditioning step before CSC solidification or slurry dewatering and determines whether ABTS should be included for each application.

## 2 Materials and methods

### 2.1 Materials

#### 2.1.1 Soil materials

Artificial soil was prepared on a dry-mass basis from 18% 325-mesh quartz-rich silt, 27% kaolin, 45% calcium bentonite, and 10% commercial humic acid (CAS 1415-93-6). The dry constituents were blended until visually uniform, hydrated with deionized water, and sealed for at least 48 h before testing. The proportions of constituents and the resulting elemental composition are summarized in Table 1, with SiO_2_ and Al_2_O_3_ as the predominant oxides. The soil had a liquid limit of 57.68%, a plastic limit of 21.43%, an optimum water content of 22%, and a maximum dry density of 1.62 g cm^−3^, the liquid limit decreased to 42.63% after oven drying, giving a ratio less than 0.75, which satisfies the ASTM organic-soil criterion. A prepared sample of soil was characterized by X-ray diffraction (XRD) using an Ultima IV diffractometer, and the pattern was analyzed using JADE 9. The dominant reflections were assigned to quartz (Figure 1). According to Unified Soil Classification System (ASTM D2487-17) the material was classified as organic clay, denoted OH throughout this paper. The artificial soil provided a reproducible humic-rich model system for comparing the tested treatment routes. The same solids formulation was dispersed for the dewatering experiments. The initial slurry water content was 383.9% on a dry-mass basis, the liquidity index was 10.0, the bulk density was 1.08 g cm^−3^, and the nominal humic-acid addition was 10% of the dry-solids mass. At this water content and density, 100 mL of slurry contained approximately 22.5 g dry solids.

**TABLE 1:** Reported oxide composition of the artificial OH soil (wt%).

Al2O3	SiO2	Fe2O3	CaO	MgO	K2O	Na2O	TiO2	Others
27.06	62.52	2.63	2.57	1.59	0.26	0.29	0.17	2.91

**FIGURE 1:**
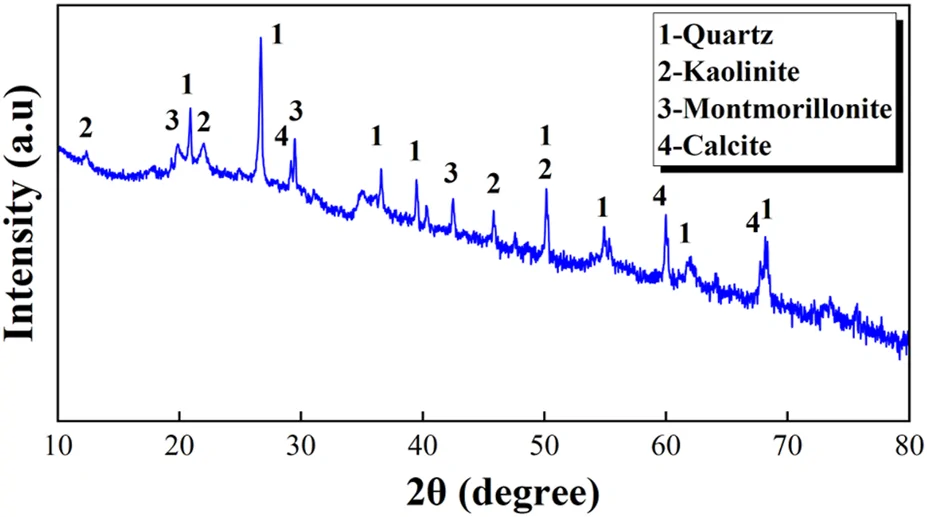
XRD pattern of the artificial OH soil.

#### 2.1.2 CSC curing agent

CSC denotes the self-developed composite curing agent used in this study. It consisted of carbide residue (CCR), gypsum, and Portland cement at a dry-mass ratio of 20:1:1. The formulation was selected from preliminary tests. CSC was added at 8% of the dry OH-soil mass in every solidification group, including the control group. CCR supplied the dominant Ca-rich alkaline phase, gypsum supplied sulfate, and Portland cement supplied hydraulic phases for development of the Ca-rich cemented matrix. The available XRF compositions of the principal OH and CSC constituents are listed in Table 2.

**TABLE 2:** Chemical composition of selected OH and CSC constituents (wt%).

Material	Al_2_O_3_	SiO_2_	Fe_2_O_3_	CaO	MgO	K_2_O	Na_2_O	TiO_2_	Others
Kaolin	41.50	53.20	0.86	0.19	0.11	0.64	0.29	1.00	2.21
Bentonite	14.80	67.00	3.79	5.01	3.19	1.00	0.22	0.15	4.84
Quartz-rich silt	2.96	96.25	0.50	0.15	—	0.11	—	—	0.03
CCR	2.06	3.79	0.70	91.65	0.11	0.04	0.08	0.15	1.27
Cement	4.33	22.34	3.6	65.10	1.31	0.82	0.49	—	0.81

#### 2.1.3 Preparation of solution and flocculant

Commercial laccase (LAC; CAS 80498-15-3), derived from *Aspergillus oryzae* by submerged fermentation, and ABTS diammonium salt (CAS 30931-67-0; purity >98%) were supplied by Hefei Yingya Biotechnology Co., Ltd. The supplier reported a laccase activity of ≥10,000 U·g^−1^. According to the supplier specification, one unit (U) of activity was defined as the amount of enzyme required to oxidize 1 μmol of guaiacol per minute at approximately pH 4.5. Laccase dosage was calculated gravimetrically from this nominal activity. Accordingly, the reported dosages of 20,000 and 40,000 U·kg^−1^ dry solids represent nominal activity-equivalent dosages based on the mass of commercial enzyme preparation added. Residual laccase activity in the soil matrix during the 24-h pretreatment was not independently quantified. Polyaluminum chloride (PAC; approximately 30% Al_2_O_3_, basicity 75%) was used as the inorganic coagulant, while cationic polyacrylamide (cPAM; molecular weight approximately 1.0 × 10^6^ Da) and carboxymethyl chitosan (CMCS; carboxylation degree ≥80%) were used as polymeric conditioners.

For slurry conditioning, PAC, CMCS, and cPAM stock solutions were freshly prepared in deionized water before use. PAC was prepared at 10% (w/v), whereas CMCS and cPAM were prepared at 1% (w/v) by gradually dispersing the polymer powders into deionized water under continuous stirring until complete dissolution. The working dosages were 9.6, 0.9, and 0.8 g kg^−1^ dry solids for PAC, CMCS, and cPAM, respectively, corresponding to 2.16, 2.03, and 1.80 mL of stock solution per 100 mL of OH slurry. The PAC, CMCS, and cPAM dosages were adopted from our preceding optimization experiments on the same humic-rich slurry system.

Laccase was prepared as a 5% (w/v) stock solution in 0.1 M acetate–sodium acetate buffer at pH 4.5–5.0 and applied at a nominal activity-equivalent dosage of 20,000 U kg^−1^ dry solids, calculated from the supplier-reported activity. ABTS was prepared at 1 mg mL^−1^ in the same buffer and applied at approximately 27.1 mg kg^−1^ dry solids. The ABTS dosage was selected from preliminary LAC–ABTS formulation tests and was subsequently maintained as a fixed mediator dosage throughout the comparative experiments.

### 2.2 Testing methods

#### 2.2.1 Experimental design

The experimental program consisted of four parts (Figure 2; Table 3). First, a laccase-dosage experiment compared 20,000 and 40,000 U kg^−1^ dry soil to select the dose used in subsequent solidification tests. Second, an experiment compared LAC, LAC + ABTS, and LAC + ABTS + CMCS pretreatments to determine how stepwise addition of the mediator and biopolymer affected subsequent CSC stabilization. In both solidification modules, the soils were pretreated for 24 h before incorporation of 8% CSC. Third, bench-scale conditioning and vacuum-filtration tests compared PAC–CMCS and PAC–CPAM based materials with or without biochemical pretreatment, using supernatant turbidity, zeta potential, filtrate turbidity, and cake water content as the principal response variables. Finally, the three selected conditioning schemes were evaluated using one 2-L geotextile tube per formulation.

**FIGURE 2:**
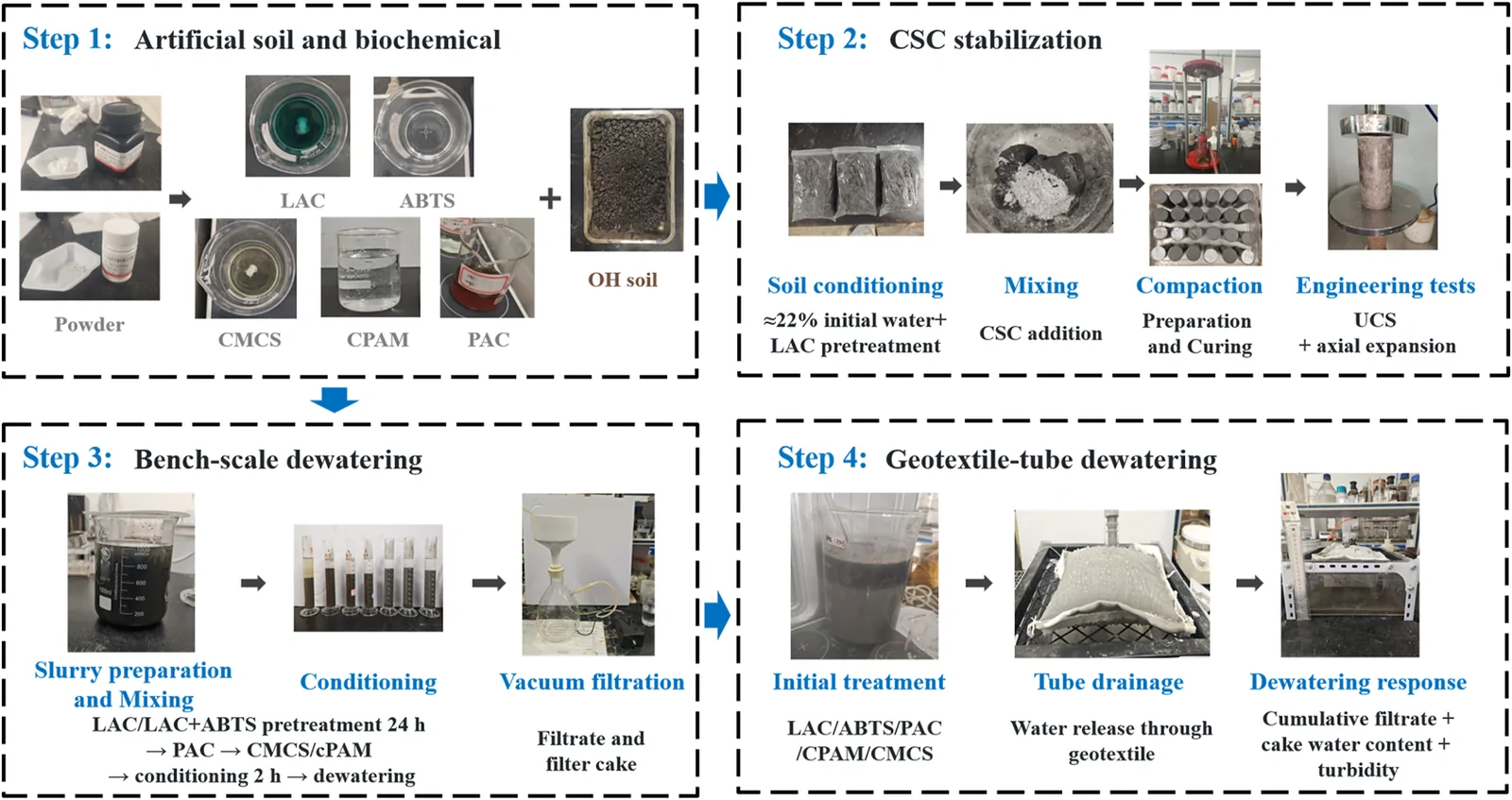
Experimental workflow.

**TABLE 3:** Experimental modules, treatment groups, and principal response variables.

Experimental module	Treatment groups	Treatment sequence	Main measurements
Laccase-dosage	OH; OH + LAC1 (20,000 U kg^−1^); OH + LAC2 (40,000 U kg^−1^)	Pretreatment solution (24 h) → 8% CSC	UCS and axial expansion at 3, 7, and 14 days
Solidification	OH; LAC; LAC + ABTS; LAC + ABTS + CMCS	Biochemical pretreatment (24 h) → 8% CSC	UCS and axial expansion at 3 and 14 days
Bench-scale dewatering	OH; PAC + CMCS; LAC + PAC + CMCS; LAC + ABTS + PAC + CMCS; PAC + cPAM; LAC + PAC + cPAM; LAC + ABTS + PAC + cPAM	Where applicable, LAC/LAC + ABTS pretreatment (24 h) → PAC → CMCS or cPAM → conditioning (2 h)	Supernatant turbidity, zeta potential, filtrate turbidity, cake water content
Geotextile-tube dewatering	LAC + ABTS + PAC + CMCS; PAC + CMCS; PAC + cPAM	Biochemical pretreatment (24 h) → PAC → CMCS or cPAM → conditioning (2 h) → tube drainage	Cumulative filtrate volume, cake water content, filtrate turbidity, conductivity

#### 2.2.2 Enzymatic pretreatment and sample preparation

Each batch was prepared using 1.0 kg of dry OH soil. For all solidification samples, the combined amount of deionized water and treatment solution was adjusted to obtain a final water content of approximately 22% before CSC addition. In the laccase-dosage test, three groups were prepared: OH control, OH + LAC1 containing 20,000 U kg^−1^ laccase, and OH + LAC2 containing 40,000 U kg^−1^ laccase. In the subsequent solidification test, LAC was fixed at 20,000 U kg^−1^, and the groups comprised OH, LAC, LAC + ABTS, and LAC + ABTS + CMCS. The control received the same total volume of 0.1 M acetate–sodium acetate buffer (pH 4.5–5.0) as the corresponding biochemical treatment group, but without LAC or ABTS, so that the added liquid volume and buffer conditions were matched among treatments.

Following pretreatment, CSC was incorporated at 8% of the dry-soil mass (80 g kg^−1^) and thoroughly homogenized with the soil. The mixtures were statically compacted in three equal layers into cylindrical moulds with a diameter of 50 mm and a height of 100 mm, with the surface of each layer lightly scarified before placement of the subsequent layer. After demolding, the specimens were cured at 20 ℃ and approximately 95% relative humidity for 3, 7, or 14 days. UCS was measured at a loading rate of 1 mm min^−1^, and axial expansion was determined from changes in specimen height measured with a vernier caliper.

#### 2.2.3 Dewaterability assessment

OH slurry was prepared at a liquidity index of 10 and divided into 100-mL aliquots. Seven conditioning routes were evaluated: untreated OH, PAC + CMCS, LAC + PAC + CMCS, LAC + ABTS + PAC + CMCS, PAC + cPAM, LAC + PAC + cPAM, and LAC + ABTS + PAC + cPAM. Where applicable, LAC and/or ABTS were introduced during a 24-h biochemical pretreatment stage. PAC was subsequently added under rapid mixing at 200 rpm for 60 s, followed by CMCS or cPAM under slow mixing at 40 rpm for 120 s. The conditioned suspensions were then maintained for 2 h before subsequent characterization and dewatering tests. Reagent dosages were those specified in Section 2.1.3.

After the 2-h conditioning period, supernatant turbidity was measured using a portable turbidimeter (Leici WZB-172E), and the zeta potential of residual suspended particles was determined using a ZetaPlus analyzer (Brookhaven, United States). Dewatering was then assessed by vacuum filtration using a Büchner funnel at a constant vacuum pressure of 0.06 MPa for 120 s. Filtrate turbidity and final cake water content were determined after filtration. Cake water content was calculated on a dry-mass basis after oven drying at 70 ℃. Each bench-scale group contained three recorded replicates, and values are reported as mean ± sample standard deviation.

#### 2.2.4 Geotextile-tube dewatering

Geotextile-tube dewatering was evaluated using 2 L of OH slurry with an initial water content of 383.9% and a liquidity index of 10. The slurry was placed in 20 cm × 20 cm geotextile tubes positioned on a permeable rack above acrylic collection boxes. Three conditioning schemes were compared: LAC + ABTS pretreatment followed by PAC + CMCS, PAC + CMCS without biochemical pretreatment, and PAC + cPAM without biochemical pretreatment.

For the LAC + ABTS route, the slurry was pretreated with LAC and ABTS for 24 h. PAC and CMCS were then sequentially added using the same coagulation/flocculation procedure as in the bench-scale tests, followed by a 2-h conditioning period before transfer to the geotextile tube. The PAC + CMCS and PAC + cPAM routes underwent the corresponding PAC–polymer conditioning procedure followed by the same 2-h conditioning period before tube drainage. Cumulative filtrate volume was recorded from 5 min to 30 h. At the end of the 30-h drainage period, three parallel subsamples were collected from the central region of each dewatered cake. The water content of each subsample was determined separately on a dry-mass basis after oven drying at 70 ℃, and the mean of the three measurements was reported as the central-region cake water content. Collection-box filtrate turbidity, turbidity after secondary settling, and conductivity were also determined. One geotextile tube was tested for each formulation. Multiple endpoint measurements collected from the same tube were treated as within-tube subsamples rather than independent experimental replicates. The recorded and calculable system-level quantities for the three geotextile-tube tests are summarized in Supplementary Table S1.

### 2.3 Microstructural and chemical characterization

#### 2.3.1 DOC and UV–Vis characterization of organic matter

DOC analysis was conducted on three biochemical-pretreatment groups, namely, untreated OH, OH + LAC, and OH + LAC + ABTS, before PAC–polymer conditioning, to isolate changes in the extractable organic-carbon pool associated with laccase and mediator treatment. UV–Vis analysis was conducted on extracts from untreated OH, PAC + CMCS, LAC + PAC + CMCS, and LAC + ABTS + PAC + CMCS to characterize differences in the UV-absorbing organic fraction after the complete CMCS-based conditioning sequence. Samples were extracted with ultrapure water at a liquid-to-solid ratio of 10 mL g^−1^ dry solids for 24 h on an orbital shaker at 150 rpm. After extraction, the suspensions were allowed to settle for 10 min without centrifugation and were then filtered through pre-rinsed 0.45-μm polyethersulfone (PES) membrane filters. The same extraction and filtration procedure was applied to all treatment groups. The filtrates were analyzed for dissolved organic carbon (DOC) using a Shimadzu TOC-L total organic carbon analyzer. UV–Vis absorption spectra were recorded over 200–800 nm using a Shimadzu UV-3600 spectrophotometer with a 1-cm quartz cuvette. Ultrapure water subjected to the same settling and filtration procedure was used as the procedural blank.

#### 2.3.2 SEM, EDS, and XRD characterization of CSC-solidified samples

Microstructural and mineralogical characterization was conducted on three groups of CSC-solidified OH soil cured for 14 days: OH + CSC, OH + LAC + CSC, and OH + LAC + ABTS + CSC. All specimens contained 8% CSC and were prepared and cured under identical conditions. Representative material was collected from the interior of each specimen to minimize surface-drying and boundary effects.

For SEM observation, the collected specimens were trimmed into approximately 1-cm fragments, rapidly frozen in liquid nitrogen, and freeze-dried under vacuum for 24 h. The dried samples were mounted and sputter-coated before imaging using a scanning electron microscope (MIRA4, TESCAN, Czech Republic). SEM images were acquired at matched magnifications to compare particle contacts, pore morphology, surface coverage, and the continuity of cementitious products among the three groups. Local elemental composition and spatial distribution were further examined using an energy-dispersive X-ray spectroscopy system (Ultim Max 65, Oxford Instruments, United Kingdom) coupled to the SEM. One representative EDS area was analyzed from representative interparticle regions within the interior of the specimen at comparable SEM magnifications. Regions showing obvious edge effects, preparation artifacts, or isolated atypical particles were excluded from the comparison. EDS results were interpreted as local compositional information rather than bulk elemental concentrations.

For XRD analysis, material collected from the interior of the 14-day specimens was gently ground to a fine powder and analyzed using an Ultima IV diffractometer. Diffraction patterns were recorded over a 2θ range of 10°–80° at 2° min^−1^ and interpreted using JADE 9 and standard diffraction databases. Diffraction patterns were acquired under identical conditions, and peak positions and relative intensities were compared among the three groups.

#### 2.3.3 FTIR characterization of filter cakes

FTIR spectroscopy was used to examine treatment-dependent changes in vacuum-filtered OH cakes. Four groups were analyzed: untreated OH, OH + LAC + PAC + CMCS, OH + LAC + ABTS + PAC + CMCS, and OH + LAC + ABTS + PAC + cPAM. Representative cake material was collected after vacuum filtration, dried, and homogenized. Spectra were obtained using a Nicolet Summit X spectrometer (Thermo Fisher Scientific, United States) over 4,000–400 cm^−1^, baseline-corrected, and compared under identical acquisition conditions. The plotted spectra were vertically offset for clarity. Peak positions and band shapes were examined in the O–H/N–H stretching region, aliphatic C–H region, overlapping carbonyl/amide and carboxylate regions, and the dominant Si–O band.

### 2.4 Statistical analysis

Unless otherwise stated, quantitative experiments were conducted using three independent replicates (n = 3), and data are presented as mean ± standard deviation (SD). For the solidification experiments, the effects of treatment formulation, curing age, and their interaction on UCS and axial expansion were evaluated using two-way analysis of variance (ANOVA), followed by Tukey’s honestly significant difference (HSD) test for pairwise comparisons among treatments within each curing age. For bench-scale dewatering indices and DOC measurements, differences among treatment groups were evaluated using one-way ANOVA followed by Tukey’s HSD test. Statistical significance was defined at P < 0.05.

The geotextile-tube experiment was conducted using one independent tube per formulation (n = 1); therefore, no inferential statistical comparison was performed among the geotextile treatments. Multiple measurements or subsamples obtained from the same tube were treated as within-tube measurements rather than independent replicates. Geotextile-tube results are consequently presented descriptively as an engineering-scale comparison. Exact ANOVA P values and Tukey-adjusted pairwise P values are provided in Supplementary Tables S2–S6.

## 3 Results

### 3.1 Effect of laccase dosage on CSC solidification

The UCS of all 8% CSC-treated specimens increased with curing age, while laccase pretreatment enhanced later-age strength development (Figure 3). The control group (OH) exhibited UCS values of 205.6, 332.6, and 434.9 kPa at 3, 7, and 14 days, respectively. For OH + LAC1, corresponding to a laccase dosage of 20,000 U kg^−1^, the UCS increased to 233.3, 488.6, and 841.4 kPa, representing increases of 13.5%, 46.9%, and 93.5% relative to OH. For OH + LAC2, corresponding to 40,000 U kg^−1^, the UCS values were 211.6, 433.6, and 818.3 kPa, representing increases of 2.9%, 30.4%, and 88.1%, respectively. UCS did not differ significantly among the three groups at 3 days. At 7 days, both OH + LAC1 and OH + LAC2 were significantly stronger than OH (P = 0.000309 and 0.00321, respectively), and OH + LAC1 was slightly but significantly higher than OH + LAC2 (P = 0.0495). At 14 days, both LAC-treated groups remained significantly stronger than OH (P = 0.000539 and 0.000743), whereas OH + LAC1 and OH + LAC2 did not differ significantly (P = 0.897). At each curing age, increasing the laccase dosage from OH + LAC1 to OH + LAC2 did not yield a higher mean UCS, indicating that the strengthening response did not increase proportionally with enzyme dosage within the tested range. Accordingly, the dosage used in OH + LAC1 was selected for the subsequent experiments. Axial expansion showed no monotonic relationship with laccase dosage (Figure 3). OH exhibited expansion values of 2.56%, 2.20%, and 2.37% at 3, 7, and 14 days, respectively. The corresponding values were 2.45%, 2.73%, and 2.86% for OH + LAC1 and 2.31%, 2.83%, and 2.53% for OH + LAC2. Thus, the pronounced increase in UCS induced by laccase pretreatment was not accompanied by a consistent reduction in axial expansion. No significant pairwise differences in axial expansion were detected among treatments within individual curing ages (Tukey HSD, P > 0.05).

**FIGURE 3:**
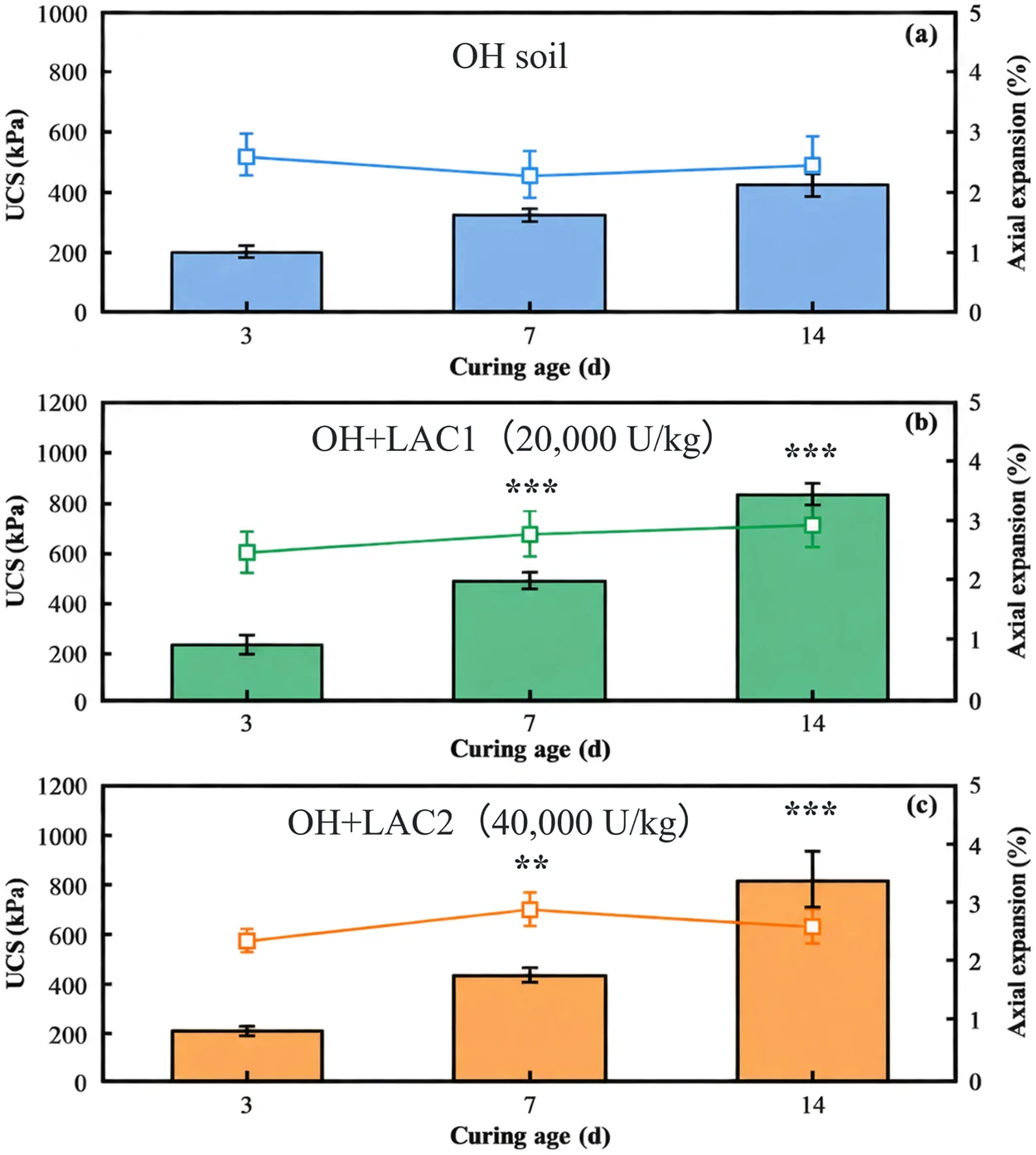
Effect of laccase dosage on UCS and axial expansion of 8% CSC-stabilized OH soil after 3, 7, and 14 days of curing. **(a)** OH, control group; **(b)** OH + LAC1, 20,000 U kg^−1^ laccase; and **(c)** OH + LAC2, 40,000 U kg^−1^ laccase. Data are presented as mean ± SD (n = 3 independent replicates). Asterisks above the UCS bars indicate significant differences from the corresponding OH control at the same curing age according to Tukey’s HSD test (**P < 0.01, ***P < 0.001).

### 3.2 Effects of ABTS and CMCS on laccase-assisted CSC solidification

The effects of ABTS and CMCS on laccase-assisted CSC solidification were evaluated by sequentially introducing these components into the LAC treatment (Figure 4). At 3 days, the OH, LAC, and LAC + ABTS groups showed similar mean UCS values of 246.3, 250.7, and 245.5 kPa, respectively, whereas the LAC + ABTS + CMCS group exhibited a lower UCS of 193.7 kPa. The LAC + ABTS + CMCS group was significantly lower than the other three groups at 3 days (Tukey HSD, P < 0.05). The differences became much more pronounced after 14 days of curing. LAC alone produced the highest UCS of 797.4 kPa, corresponding to a 94.2% increase relative to OH (410.6 kPa). The addition of ABTS reduced the 14-day UCS to 630.6 kPa, although it remained 53.6% higher than OH. Further addition of CMCS decreased the UCS to 518.0 kPa, corresponding to a 26.2% increase over OH. At 14 days, all four formulations differed significantly, following the order LAC > LAC + ABTS > LAC + ABTS + CMCS > OH (P < 0.05). Overall, laccase pretreatment markedly enhanced later-age strength development, whereas the additional incorporation of ABTS and CMCS progressively diminished this strengthening effect.

**FIGURE 4:**
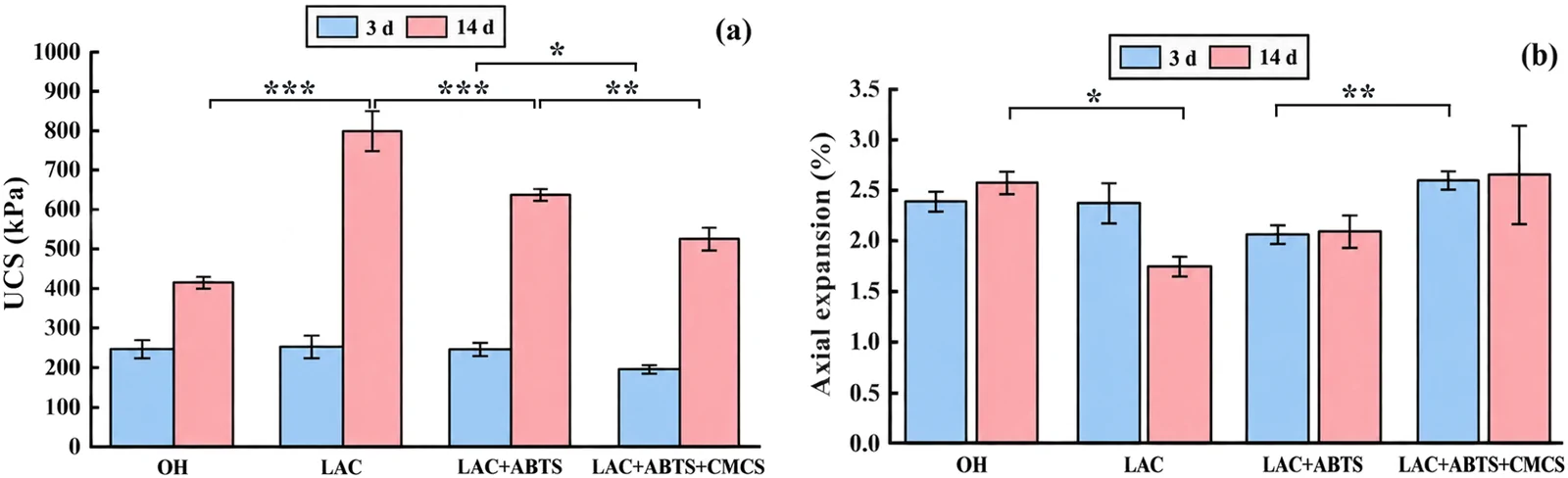
Effects of ABTS and CMCS on laccase-assisted CSC solidification of OH soil. **(a)** Unconfined compressive strength (UCS) after 3 and 14 days of curing. **(b)** Axial expansion after 3 and 14 days of curing. Treatments included OH, LAC, LAC + ABTS, and LAC + ABTS + CMCS. Data are presented as mean ± SD (n = 3). Asterisks indicate significant Tukey HSD comparisons within the same curing age (*P < 0.05, **P < 0.01, ***P < 0.001)

Axial expansion also depended on formulation, but the group ranking changed with curing age (Figure 4b). At 3 days, expansion was 2.38% for OH, 2.36% for LAC, 2.06% for LAC + ABTS, and 2.59% for LAC + ABTS + CMCS, with the lowest value observed for LAC + ABTS. By 14 days, however, LAC showed the lowest expansion of 1.74%, compared with 2.58% for OH, 2.08% for LAC + ABTS, and 2.64% for LAC + ABTS + CMCS. Thus, among the tested formulations, LAC alone combined the highest 14-day UCS with the lowest 14-day axial expansion. The addition of ABTS, and particularly the subsequent incorporation of CMCS, did not provide an additive benefit to the later-age CSC solidification performance.

### 3.3 Flocculation and dewatering performance

Bench-scale conditioning markedly altered the clarification and dewatering behavior of the OH slurry (Figure 5). Treatment formulation significantly affected supernatant turbidity, zeta potential, vacuum-filtrate turbidity, and cake water content (one-way ANOVA, P < 0.001). The untreated slurry exhibited a supernatant turbidity of 30.13 NTU and a zeta potential of −15.53 mV. After PAC–polymer conditioning, zeta potentials shifted to a relatively narrow range of −6.73 to −8.65 mV, while supernatant turbidity decreased substantially across all formulations. All PAC-containing formulations differed significantly from untreated OH in zeta potential, whereas no significant differences were detected among the PAC-containing formulations themselves. Within the CMCS-based branch, PAC + CMCS reduced supernatant turbidity to 6.71 NTU, whereas LAC + PAC + CMCS produced a significantly higher value of 10.06 NTU. Further incorporation of ABTS in the LAC + ABTS + PAC + CMCS route significantly reduced turbidity to 3.73 NTU, the lowest value among the CMCS-based formulations. Notably, D2 and D3 exhibited similar zeta potentials of −7.12 and −6.89 mV, respectively, despite their markedly different supernatant turbidities, indicating that charge reduction alone did not determine clarification performance.

**FIGURE 5:**
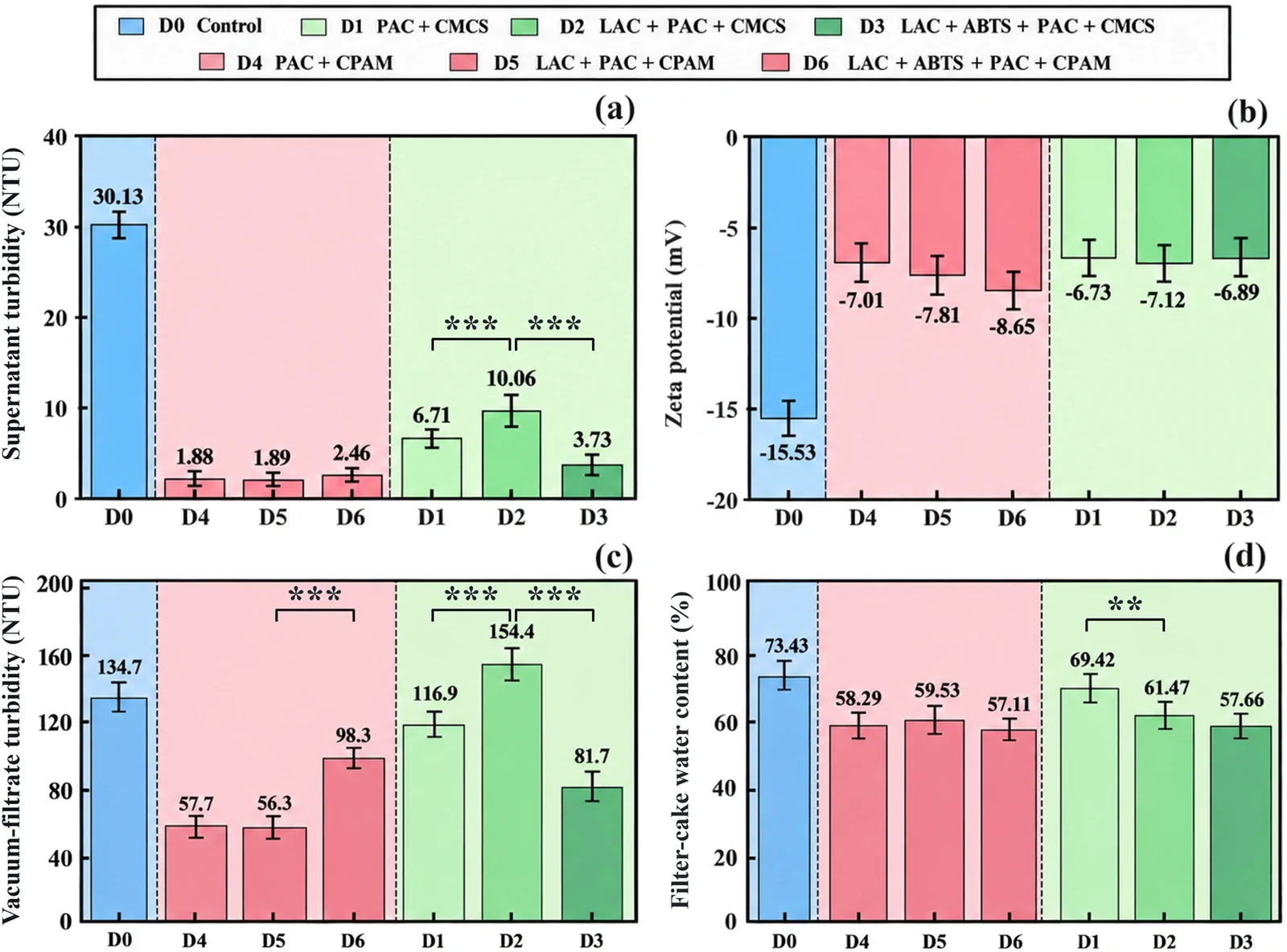
Bench-scale flocculation and dewatering performance of OH slurry. **(a)** Supernatant turbidity, **(b)** zeta potential, **(c)** vacuum-filtrate turbidity, and **(d)** filter-cake water content. D0, control; D1, PAC + CMCS; D2, LAC + PAC + CMCS; D3, LAC + ABTS + PAC + CMCS; D4, PAC + cPAM; D5, LAC + PAC + cPAM; and D6, LAC + ABTS + PAC + cPAM. Data are presented as mean ± SD (n = 3). Asterisks indicate selected Tukey HSD comparisons between sequential formulations (**P < 0.01, ***P < 0.001).

The differences among the CMCS-based formulations became more pronounced after vacuum filtration. PAC + CMCS produced a filtrate turbidity of 116.9 NTU and a cake water content of 69.42%. Introducing LAC before PAC + CMCS reduced cake water content to 61.47%, but increased filtrate turbidity to 154.4 NTU. In contrast, LAC + ABTS + PAC + CMCS significantly reduced filtrate turbidity to 81.7 NTU, while the mean cake water content further decreased to 57.66%, although the latter difference from LAC + PAC + CMCS was not significant. Thus, mediator addition improved filtrate clarity relative to the corresponding LAC + PAC + CMCS route, even though the two formulations exhibited similar zeta potentials.

The cPAM-based formulations generally provided clearer liquid phases than the CMCS-based formulations. PAC + cPAM yielded a supernatant turbidity of 1.88 NTU, a vacuum-filtrate turbidity of 57.7 NTU, and a cake water content of 58.29%. LAC + PAC + cPAM produced similar supernatant turbidity at 1.89 NTU and the lowest filtrate turbidity of 56.3 NTU, with a cake water content of 59.53%. Adding ABTS to the LAC + PAC + cPAM route reduced cake water content to 57.11%, but increased supernatant and filtrate turbidity to 2.46 and 98.3 NTU, respectively. The increase in filtrate turbidity was significant, whereas the changes in supernatant turbidity and cake water content were not. Overall, the ranking of the seven formulations differed among zeta potential, supernatant turbidity, filtrate turbidity, and cake water content.

### 3.4 Geotextile-tube dewatering performance

The three conditioning schemes showed distinct drainage behaviors during geotextile-tube dewatering (Figure 6). Starting from an OH slurry with an initial water content of 383.9%, PAC + CPAM exhibited the fastest initial drainage, releasing 725 mL of filtrate within the first 5 min, compared with 520 mL for LAC + ABTS + PAC + CMCS and 150 mL for PAC + CMCS. Although PAC + cPAM released the greatest filtrate volume during the first 5 min, LAC + ABTS + PAC + CMCS subsequently achieved the greatest cumulative water release and maintained the highest final filtrate volume through the end of the test. At 30 h, the cumulative filtrate volume reached 1702.8 mL for LAC + ABTS + PAC + CMCS, compared with 1,340 mL for PAC + CMCS and 1,392 mL for PAC + CPAM. The final cumulative filtrate volume was therefore 27.1% and 22.3% greater than that of PAC + CMCS and PAC + cPAM, respectively.

**FIGURE 6:**
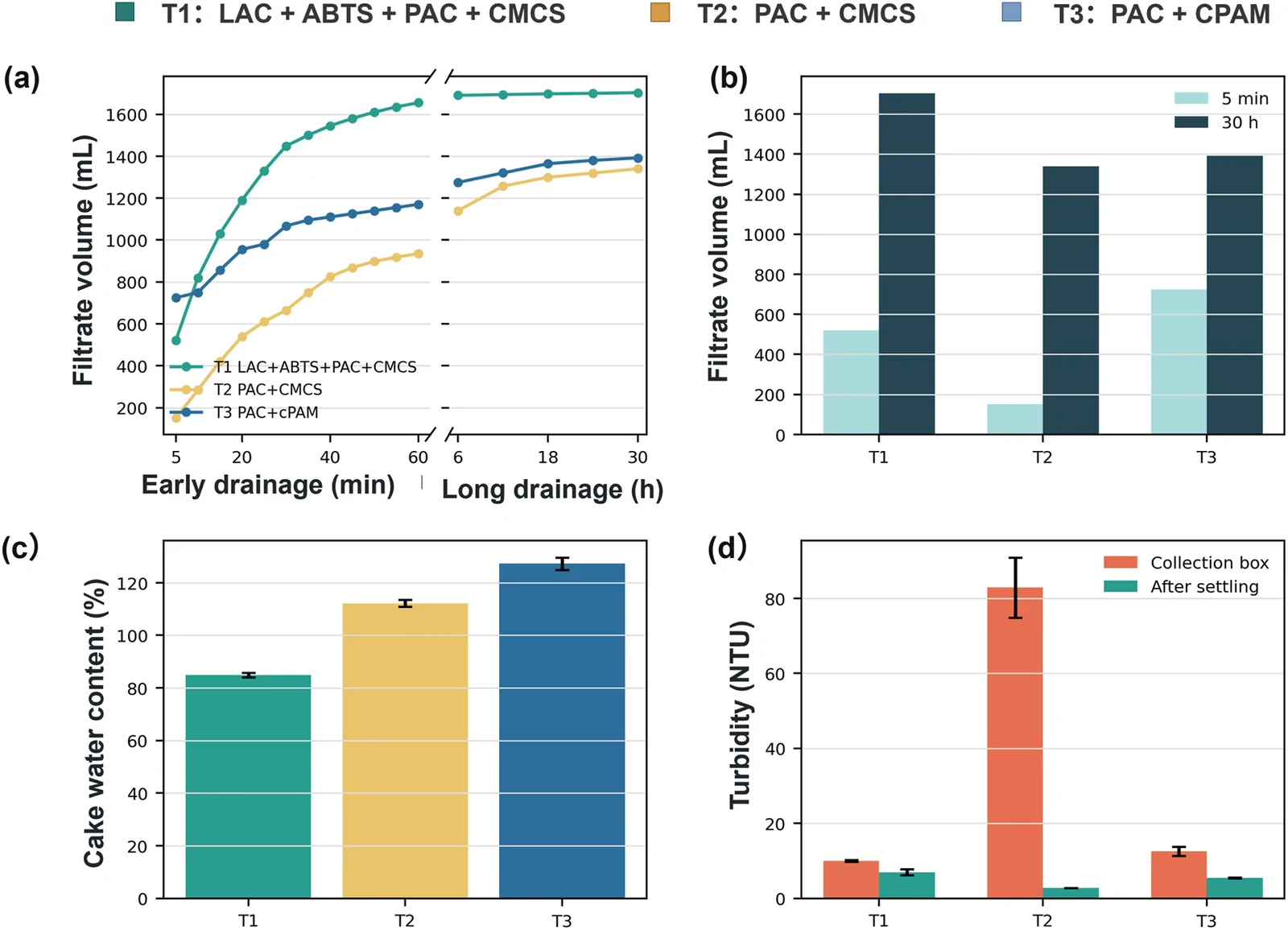
Geotextile-tube dewatering performance. **(a)** Cumulative filtrate volume during 30 h drainage; **(b)** filtrate volumes at 5 min and 30 h; **(c)** central-region cake water content; and **(d)** collection-box filtrate turbidity and turbidity after secondary settling. One independent geotextile tube was tested per formulation (n = 1). Error bars represent the SD of three central-region subsamples from the same tube and do not represent independent experimental replicates.

The enhanced cumulative drainage was accompanied by a lower central-region cake water content. At the end of the 30-h test, the cake water content was 84.94% for LAC + ABTS + PAC + CMCS, substantially lower than 112.19% for PAC + CMCS and 127.23% for PAC + CPAM. The differences relative to the two conventional conditioning schemes were 27.25 and 42.29 percentage points, respectively. The directly collected filtrate from LAC + ABTS + PAC + CMCS also remained relatively clear, with a turbidity of 9.99 NTU, compared with 82.86 NTU for PAC + CMCS and 12.53 NTU for PAC + CPAM. After secondary settling, the corresponding turbidity values decreased to 6.99, 2.79, and 5.49 NTU. Among the three tested tubes, LAC + ABTS + PAC + CMCS showed the greatest cumulative water release and the lowest central-region cake water content while maintaining relatively low turbidity in the directly collected filtrate. In contrast, PAC + CMCS produced highly turbid initial filtrate that clarified markedly during subsequent settling. Endpoint conductivity was 3,875 μS cm^−1^ for LAC + ABTS + PAC + CMCS, 3,685 μS cm^−1^ for PAC + CMCS, and 3,369 μS cm^−1^ for PAC + CPAM.

### 3.5 Characteristics of extractable organic matter

Laccase pretreatment progressively decreased the DOC concentration of the OH extracts (Figure 7a). DOC decreased from 40.50 mg C L^−1^ in untreated OH to 29.07 mg C L^−1^ after LAC treatment, corresponding to a reduction of 28.2%. Addition of ABTS further decreased DOC to 20.33 mg C L^−1^, representing a reduction of 49.8% relative to untreated OH and 30.0% relative to LAC alone. Thus, the DOC concentration followed the order OH > OH + LAC > OH + LAC + ABTS. DOC differed significantly among all three groups, following the order OH > OH + LAC > OH + LAC + ABTS (Tukey HSD, P < 0.001).

**FIGURE 7:**
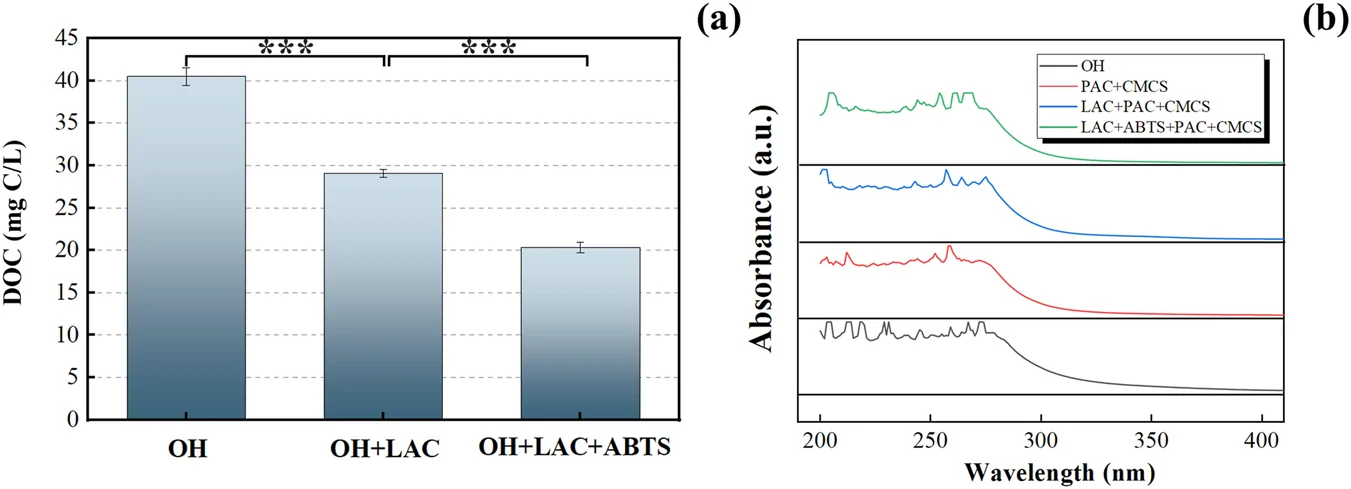
Characteristics of extractable organic matter under biochemical pretreatment and PAC–CMCS conditioning. **(a)** DOC concentrations of untreated OH, OH + LAC, and OH + LAC + ABTS extracts. **(b)** UV–Vis absorption spectra of OH, PAC + CMCS, LAC + PAC + CMCS, and LAC + ABTS + PAC + CMCS extracts. Data are presented as mean ± SD (n = 3). Asterisks indicate Tukey HSD comparisons between sequential treatments (***P < 0.001).

The UV–Vis spectra of the conditioned extracts also differed substantially among treatments (Figure 7b). Untreated OH exhibited the highest absorbance over most of the 300–400 nm region, whereas PAC + CMCS and LAC + PAC + CMCS showed intermediate spectral responses. LAC + ABTS + PAC + CMCS exhibited the lowest absorbance over most of this wavelength range. At 320, 350, and 365 nm, its absorbance was approximately 69%, 74%, and 73% lower than that of untreated OH, respectively. PAC + CMCS and LAC + PAC + CMCS showed partially overlapping spectra, with their relative absorbance varying with wavelength, whereas the LAC + ABTS + PAC + CMCS spectrum remained consistently lower over the principal comparison region.

### 3.6 Microstructural and mineralogical characterization

SEM observations showed clear treatment-dependent differences in the microstructure of the 14-day CSC-solidified specimens (Figures 8a–c). The OH + CSC specimen exhibited a relatively loose and heterogeneous structure, with visible interparticle voids and discontinuous deposits on particle surfaces. The LAC + ABTS-pretreated specimen showed a comparatively denser structure, with increased surface coverage by reaction products and fewer pronounced open voids. The most compact morphology was observed in OH + LAC + CSC, where the particle surfaces were more extensively covered by cementitious products and adjacent particles appeared to be connected by more continuous solid bridges. Although individual hydration products cannot be identified reliably from morphology alone, the progressive densification from OH + CSC to OH + LAC + ABTS + CSC and OH + LAC + CSC was consistent with the corresponding 14-day mechanical response, with LAC alone producing the most favorable cemented structure.

**FIGURE 8:**
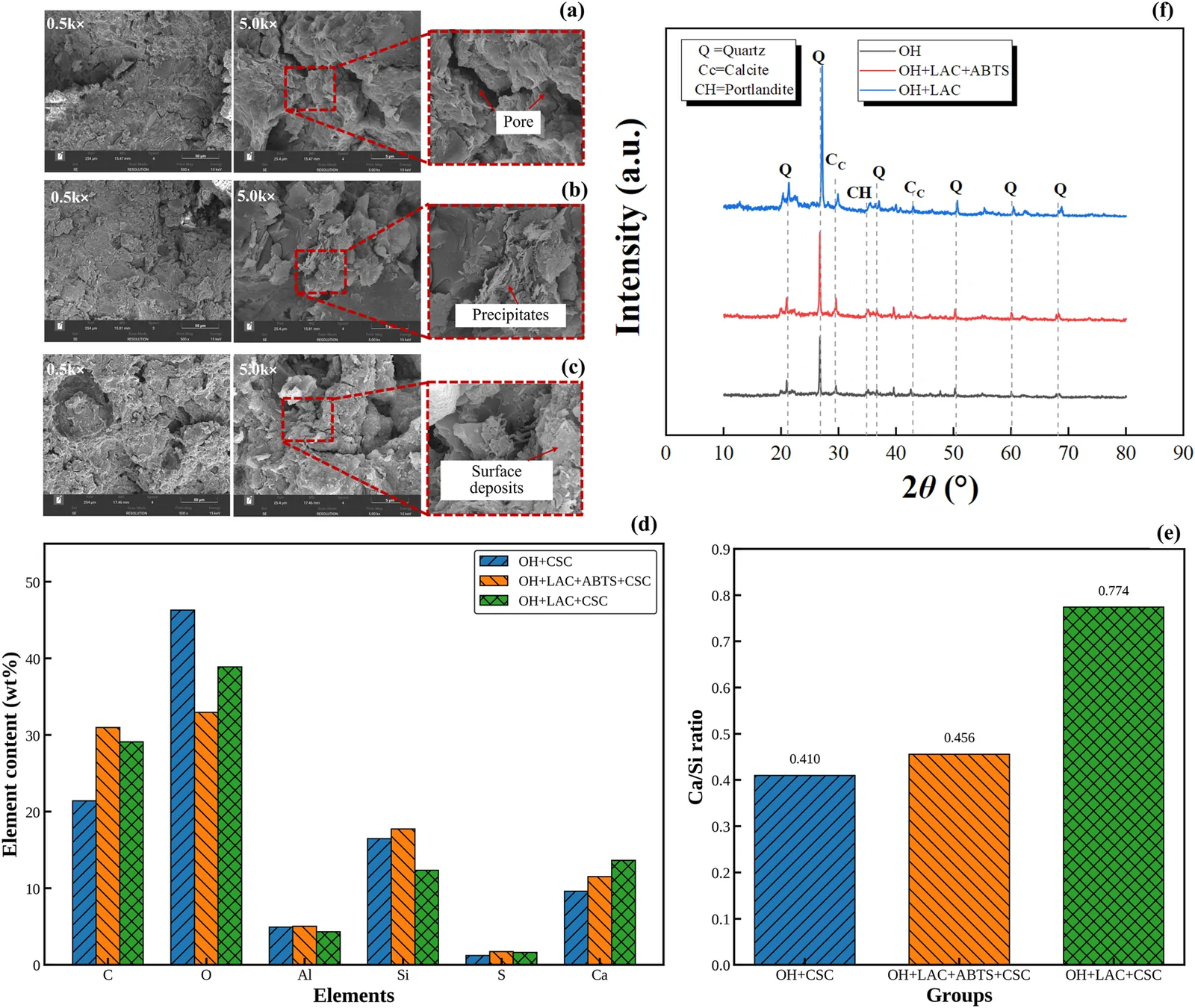
SEM–EDS–XRD characterization of 14-day CSC-solidified OH specimens. **(a–c)** SEM morphologies of OH + CSC, OH + LAC + ABTS + CSC, and OH + LAC + CSC; **(d)** EDS elemental composition; **(e)** atomic Ca/Si ratio; and **(f)** XRD patterns. Q, quartz; Cc, calcite; CH, portlandite.

Local EDS analysis further revealed differences in the elemental composition of the examined cemented regions (Figures 8d,e). The Ca content increased from 9.63 wt% in OH + CSC to 11.53 wt% in OH + LAC + ABTS + CSC and 13.65 wt% in OH + LAC + CSC. The atomic Ca/Si ratio increased from 0.410 to 0.456 and 0.774, respectively, showing the strongest relative enrichment of Ca-bearing material in the LAC-treated region. Sulfur increased from 1.23 wt% in OH + CSC to 1.74 and 1.63 wt% in the LAC + ABTS- and LAC-treated specimens, respectively. C and O varied non-monotonically and were not used to quantify organic-matter removal. As a local, semiquantitative measurement, EDS establishes local Ca enrichment rather than a bulk phase fraction. Together with SEM, these observations show a denser interparticle morphology accompanied by greater local Ca enrichment after LAC pretreatment, with an intermediate response after ABTS addition.

The XRD patterns of the three specimens showed broadly similar crystalline-phase assemblages, dominated by quartz together with calcite and portlandite reflections (Figure 8f). The characteristic quartz peaks, including the dominant reflection near 26.6°, remained at essentially unchanged diffraction positions, indicating that enzymatic pretreatment did not alter the principal aluminosilicate mineral framework. No distinct new crystalline phase was detected after LAC or LAC + ABTS pretreatment. Differences were instead observed in the relative prominence of the Ca-bearing reflections, particularly those associated with calcite and portlandite. When considered together with the local EDS observations, the XRD patterns show treatment-dependent differences in the relative prominence of existing Ca-bearing reflections, without evidence for formation of a distinct new crystalline phase. Overall, the stronger LAC-treated specimen was associated with a denser interparticle morphology and greater local Ca enrichment, while the dominant crystalline-phase assemblage remained essentially unchanged. The LAC + ABTS-treated specimen showed an intermediate microstructural response, consistent with its lower 14-day UCS and greater axial expansion than the LAC-treated specimen.

### 3.7 Functional-group characterization of filter cakes

All vacuum-filtered cakes exhibited the characteristic FTIR features of the OH mineral–organic matrix (Figure 9), including structural O–H bands at approximately 3,696 and 3,620 cm^−1^, a broad O–H/N–H stretching band at 3,425–3,435 cm^−1^, an overlapping absorption band near 1,625–1,630 cm^−1^, and a dominant Si–O stretching band at approximately 1,035 cm^−1^. The structural O–H and Si–O bands remained at essentially unchanged positions after conditioning, indicating preservation of the aluminosilicate mineral framework. In the PAC–CMCS system, the broad O–H/N–H band occurred at approximately 3,425 cm^−1^ for both OH and OH + LAC + PAC + CMCS and shifted slightly to approximately 3,430 cm^−1^ following ABTS addition. In contrast, the band near 1,630 cm^−1^ remained within a narrow range of approximately 1,625–1,628 cm^−1^. More evident treatment-dependent differences occurred in the band shapes and relative spectral features within the 1,600–1,400 cm^−1^ region, where contributions from adsorbed water, carboxylate-containing organic species, aromatic structures, and polymer-associated functionalities overlap.

**FIGURE 9:**
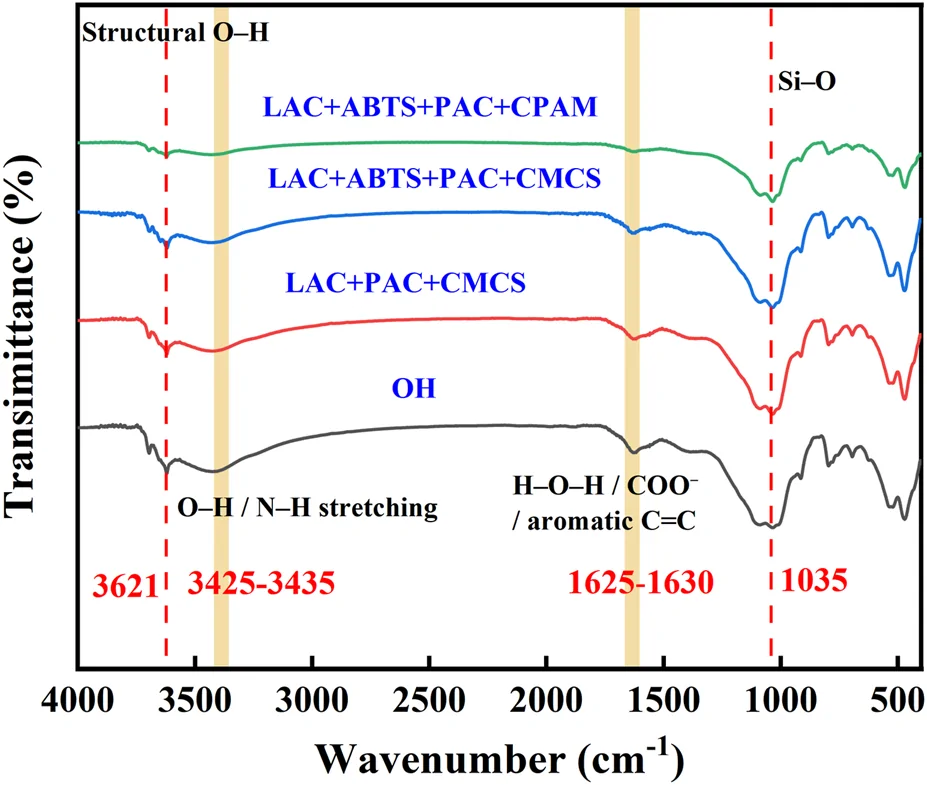
FTIR spectra of vacuum-filtered OH cakes after different conditioning treatments. Spectra show the structural O–H bands, broad O–H/N–H stretching region, overlapping 1,600–1,400 cm^−1^ region, and the dominant Si–O band. Spectra are vertically offset for clarity.

No distinct new absorption bands were generated by LAC or LAC + ABTS conditioning. The CPAM-containing cake exhibited a spectral profile different from that of the corresponding CMCS system, particularly within the 1,600–1,400 cm^−1^ region, while no isolated strong amide-I band was resolved from the overlapping OH-matrix signals. The FTIR results therefore indicate changes in the organic/polymer-associated spectral environment without evidence of alteration of the dominant aluminosilicate framework.

## 4 Discussion

### 4.1 Laccase-enhanced CSC solidification

The response of humic-rich soil to mineral binders is strongly influenced by organic material at particle and binder interfaces. Humic substances contain carboxyl, phenolic, and other oxygen-containing groups that adsorb onto mineral surfaces, interact with Ca- and Al-bearing species, retain water, and hinder contact between reactive solid phases. These effects can disrupt development of a continuous cemented skeleton in binder-treated organic soils (Cao et al., 2022; Huang et al., 2023; Yuan et al., 2025; Yu et al., 2025). In parallel, calcium-carbide-residue-based binders have emerged as a resource-efficient option for soil stabilization, making recovery of their cementing capacity in organic-rich matrices an important process objective (Yu et al., 2024; Julphunthong et al., 2024; Moazami et al., 2025). The 3-day UCS values of OH and LAC were nearly identical at 246.3 and 250.7 kPa, respectively, but the difference became marked after 14 days: UCS increased from 410.6 to 797.4 kPa, while axial expansion decreased from 2.58% to 1.74%. The dosage comparison showed the same delayed strengthening pattern. At 20,000 U kg^−1^, the UCS increase relative to its control rose from 13.5% at 3 days to 46.9% at 7 days and 93.5% at 14 days. These results indicate that LAC mainly improved strength development during subsequent CSC curing rather than contributing appreciable early strength. The small 3-day difference but large 14-day strength gain also shows that LAC itself did not provide an immediate cementitious contribution; its main effect appeared during subsequent CSC curing.

Microstructural evidence from the solidification branch supports this interpretation. The 14-day LAC + CSC specimen showed the densest matrix, the greatest local Ca content, and the highest atomic Ca/Si ratio, while XRD retained the same dominant crystalline framework but showed treatment-dependent differences in the prominence of Ca-bearing reflections. The agreement among strength, expansion, SEM morphology, and local Ca enrichment is consistent with a more favorable condition for CSC-derived cementation after LAC pretreatment.

The dosage response shows that the useful effect of laccase was not proportional to enzyme input. Increasing nominal activity from 20,000 to 40,000 U kg^−1^ did not increase UCS at any tested curing age; the 14-day values were 841.4 and 818.3 kPa, respectively. Once the accessible organic sites controlling soil–binder contact are sufficiently modified, additional enzyme does not necessarily create more mechanically effective contacts. Within the tested range, 20,000 U kg^−1^ was therefore selected as the working dosage for subsequent experiments, because increasing the nominal laccase input to 40,000 U kg^−1^ did not provide additional strength improvement.

ABTS further shows that a wider biochemical reaction range does not automatically improve solidification. Adding ABTS reduced 14-day UCS from 797.4 to 630.6 kPa and increased axial expansion from 1.74% to 2.08%; adding CMCS further reduced UCS to 518.0 kPa and increased expansion to 2.64%. The microstructural sequence was the same, with LAC alone producing the strongest densification and Ca enrichment and LAC + ABTS showing an intermediate response. CSC stabilization therefore depends on where organic interference is relieved and whether that change improves load-bearing particle–binder contacts, not simply on maximizing the extent of organic transformation. ABTS further decreased extractable DOC from 29.07 to 20.33 mg C/L, whereas the corresponding 14-day UCS decreased from 797.4 to 630.6 kPa. Thus, maximizing the reduction in extractable DOC was not equivalent to maximizing CSC solidification. The superior performance of LAC alone suggests that combined mechanical and microstructural results are consistent with the possibility that differences in the location or form of organic-matter transformation influenced subsequent particle–binder interactions. Accordingly, the present solidification results describe strength development up to 14 days and do not establish longer-term curing or durability behavior. By 14 days, the treatment-dependent differences in strength development were clearly established, providing an appropriate endpoint for comparing the solidification responses examined in this study.

### 4.2 Flocculation and dewatering performance

The governing requirements changed when the same humic-rich material was dispersed as a high-water-content slurry. In this state, treatment performance depends on destabilization of the negatively charged suspension, formation of sufficiently large and stable aggregates, retention of solids during filtration, and preservation of water-conducting pathways through the developing cake. PAC-containing treatments shifted the zeta potential from −15.53 mV to a relatively narrow range of −6.73 to −8.65 mV, indicating substantial reduction of electrostatic repulsion. However, this convergence in surface charge did not produce equivalent dewatering responses. For example, LAC + PAC + CMCS and LAC + ABTS + PAC + CMCS had similar zeta potentials of −7.12 and −6.89 mV, respectively, but showed markedly different turbidity and cake water content. Zeta-potential reduction therefore describes the initial destabilization of the suspension but does not by itself predict filtration performance (Wu et al., 2021; Liu et al., 2024). Once electrostatic repulsion is reduced, aggregate architecture, floc strength, compressibility, and pore connectivity become increasingly important in controlling dewaterability (Tan et al., 2024; Wang H.-F. et al., 2025; Zhang Z. et al., 2024).

The CMCS- and cPAM-based materials showed different response patterns. The cPAM formulations generally had lower supernatant and vacuum-filtrate turbidity, indicating stronger short-duration particle capture and clarification. Within the CMCS route, LAC lowered cake water content, and adding ABTS further reduced both cake water content and filtrate turbidity. The lowest absorbance in the 300–400 nm region was also observed for LAC + ABTS + PAC + CMCS, showing that its improved dewatering coincided with a lower UV-absorbing dissolved-organic fraction. (D’Andrilli et al., 2022; Korak and McKay, 2024; Begum et al., 2023). FTIR differences were concentrated in the broad O–H/N–H and overlapping 1,600–1,400 cm^−1^ regions, whereas the structural O–H and Si–O bands remained essentially unchanged, showing treatment-dependent changes in the organic/polymer-associated spectral environment without detectable alteration of the mineral framework.

The difference between formulations became more pronounced during geotextile-tube drainage. PAC + cPAM produced the fastest initial release of water, whereas LAC + ABTS + PAC + CMCS sustained drainage over a longer period. At 30 h, the latter produced approximately 1,703 mL of filtrate, compared with 1,340 mL for PAC + CMCS and 1,392 mL for PAC + CPAM, and yielded the lowest central-region cake water content of 84.94%. This divergence is consistent with the different hydraulic environments imposed by vacuum filtration and geotextile-tube dewatering. Vacuum filtration applies a controlled pressure gradient across a relatively thin retained layer and therefore emphasizes rapid floc capture and short-term cake resistance. In a geotextile tube, by contrast, the retained solids form a progressively thickening and deformable cake that undergoes drainage and self-consolidation over a much longer period. These results indicate that cPAM favored rapid initial drainage, whereas LAC + ABTS + PAC + CMCS better sustained water release during cake development, consistent with greater persistence of water-conducting pathways during consolidation. This scale dependence is consistent with recent studies showing that flocculation and geotextile filtration jointly control early particle capture and cake formation (Lu et al., 2024; Wang T. et al., 2025; Zhang H. et al., 2024). Long-duration drainage is then increasingly governed by cake development and consolidation (Bulut and Sengul, 2025; Karadoğan et al., 2022; Zhang et al., 2022). Similarly, the tube tests characterize drainage within the 30-h observation window; continued consolidation and longer-term drainage were not evaluated. By 30 h, drainage had slowed markedly relative to the initial stage, while clear differences among formulations in cumulative filtrate volume and central-region cake water content had been established, supporting the use of 30 h as the comparison endpoint.

The bench and geotextile-tube tests therefore captured different aspects of dewatering performance. cPAM favored short-term clarification and rapid initial drainage, whereas LAC + ABTS followed by PAC + CMCS produced the greatest cumulative drainage and the lowest central-region cake water content during prolonged tube dewatering.

### 4.3 Mechanistic basis of differential effects

The contrasting effects of ABTS on solidification and dewatering reflect the different structural requirements of the two engineering processes (Figure 10). In CSC solidification, performance depends on the development of mechanically effective particle–binder contacts during curing, whereas slurry dewatering depends on aggregate formation and the persistence of water-conducting pathways during filtration and cake consolidation. Laccase oxidizes enzyme-accessible phenolic moieties, whereas ABTS transfers oxidative equivalents to substrates beyond the immediate enzyme-accessible region (Gu et al., 2021; Wang et al., 2024; Aghaee et al., 2024). In compacted soil, CSC strength develops primarily at particle–binder contacts; in slurry, organic matter and mineral particles are dispersed throughout the aqueous phase. ABTS can therefore broaden the distribution of organic modification. Because ABTS participates in redox cycling rather than acting as an inert additive, the post-treatment system may contain both parent ABTS and mediator-derived transformed species distributed between the aqueous and solid phases. Laccase-mediated oxidation can promote cleavage, coupling, humification, and redistribution between extractable and non-extractable organic fractions (Li et al., 2022; Zhou et al., 2022; Qiao et al., 2024), therefore, the lower DOC observed after treatment reflects a shift in the extractable organic-carbon pool rather than evidence of a single degradation pathway.

**FIGURE 10:**
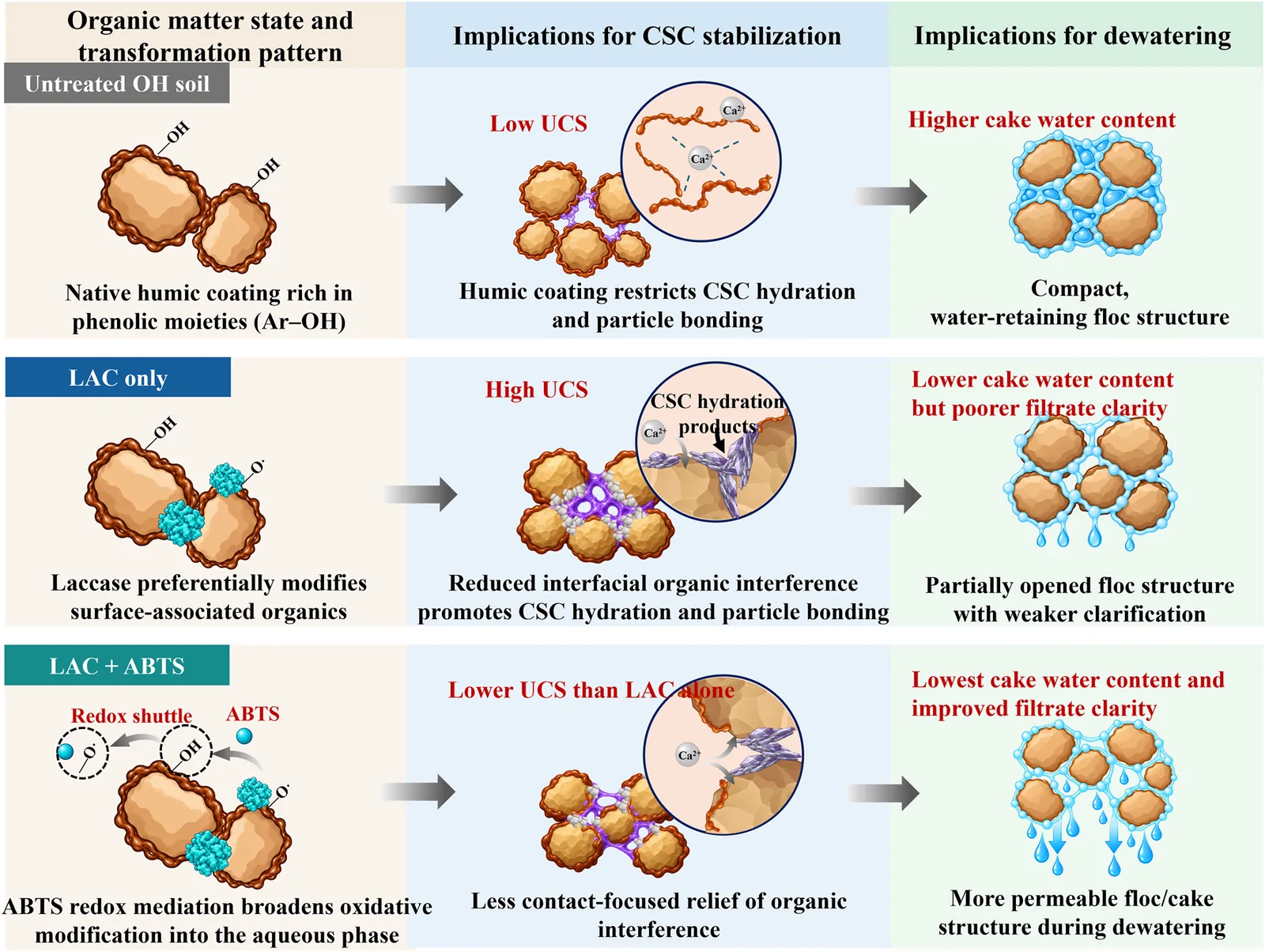
Conceptual interpretation of the differential effects of LAC and LAC + ABTS on CSC solidification and slurry dewatering.

For CSC solidification, LAC alone had the highest UCS, lowest expansion, densest cemented structure, and highest local Ca enrichment. By contrast, ABTS further lowered extractable DOC but reduced the 14-day UCS and gave an intermediate microstructure. The contrasting DOC and UCS responses show that the magnitude of extractable-DOC reduction alone did not determine CSC solidification. Differences in the location or form of organic-matter transformation may have contributed to the different particle–binder interactions. Together, these results indicate that LAC and LAC + ABTS altered the organic phase differently in ways that affected subsequent particle–binder interactions. In this conceptual framework, LAC may have produced a more favorable interfacial condition for CSC cementation, whereas ABTS-assisted treatment may have extended organic transformation over a broader fraction of the system.

In high-water slurry, broader mediator-assisted modification favored sustained dewatering. At 383.9% water content, organic matter and mineral particles were dispersed throughout the aqueous phase, providing conditions for ABTS-mediated electron transfer to extend organic modification beyond the immediate enzyme-accessible region before PAC and CMCS addition. LAC + ABTS + PAC + CMCS was not the fastest formulation during the first 5 min, but it sustained drainage and ultimately produced the greatest cumulative filtrate volume and the lowest central-region cake water content. The prolonged drainage is consistent with better preservation of water-conducting pathways during cake consolidation. ABTS therefore had opposite engineering effects in the two processes: it reduced CSC solidification performance relative to LAC alone but enhanced prolonged CMCS-based slurry dewatering. From a reuse perspective, these process-dependent formulations may also differ in the composition of the resulting liquid and solid phases, including residual enzyme/mediator species, PAC/polymer-derived constituents, and the redistribution of soluble organic and mineral fractions.

The controlled 10% humic-acid OH model isolated the treatment-dependent responses examined here; validation with natural humic-rich soils and sediments will further define the range of applicability of these formulation trends. Further measurement of residual laccase activity and post-treatment ABTS fate would clarify enzyme retention and mediator persistence, while ABTS-only, heat-inactivated-LAC, and LAC + CMCS controls would resolve the independent contributions of individual components.

## 5 Conclusion

This study demonstrated that laccase-based pretreatment produced distinct engineering responses in CSC solidification and slurry dewatering of humic-rich soil. The main conclusions are as follows.

1. Laccase pretreatment improved CSC solidification of OH soil. At 14 days, LAC treatment increased UCS from 410.6 to 797.4 kPa and reduced axial expansion from 2.58% to 1.74%. A nominal dosage of 20,000 U kg^−1^ was selected because increasing the dosage to 40,000 U kg^−1^ produced no further strength gain. Addition of ABTS and CMCS progressively reduced the solidification enhancement. Microstructural characterization showed that the LAC-treated specimen had the densest interparticle morphology and the greatest local Ca enrichment, while the dominant crystalline-phase assemblage remained essentially unchanged.
2. Slurry dewatering followed a different performance hierarchy from solidification. PAC-containing formulations effectively destabilized the negatively charged suspension, but similar zeta potentials did not produce equivalent filtrate turbidity or cake water content, showing that surface-charge reduction alone did not govern dewatering performance. In the geotextile-tube test, LAC + ABTS + PAC + CMCS produced 1,702.8 mL of cumulative filtrate at 30 h and reduced the central-region cake water content to 84.94%, compared with 1,340 mL and 112.19% for PAC + CMCS and 1,392 mL and 127.23% for PAC + cPAM. PAC + cPAM favored rapid initial drainage, whereas LAC + ABTS + PAC + CMCS produced the greatest cumulative water release and the lowest central-region cake water content during prolonged geotextile-tube dewatering.
3. The contrasting responses were process-dependent. For CSC solidification, LAC alone gave the highest UCS and lowest axial expansion, whereas adding ABTS further lowered extractable DOC but reduced the 14-day strength benefit. For slurry dewatering, adding ABTS to the LAC + PAC + CMCS system improved filtrate clarity and reduced cake water content in the bench-scale test, while LAC + ABTS followed by PAC + CMCS produced the greatest cumulative drainage and the lowest central-region cake water content in the geotextile-tube test. Thus, the enzyme–mediator combination should be selected according to the intended engineering process rather than treated as universally additive.

## Data Availability

The raw data supporting the conclusions of this article will be made available by the authors, without undue reservation.
