# Supplementary material for: Differential effects of ABTS-assisted laccase pretreatment on solidification and dewatering of humic-rich soil
Source: Front Bioeng Biotechnol. 2026 Oct 19;14:1996007. doi: 10.3389/fbioe.2026.1996007 (PMC13724566; doi:10.3389/fbioe.2026.1996007)
Supplement: Supplementary file 1 [file Table1.docx]

**Table S1. Recorded and calculable quantities for the geotextile-tube dewatering tests.**

| **Quantity** | **LAC+ABTS+PAC+CMCS** | **PAC+CMCS** | **PAC+cPAM** |
| --- | --- | --- | --- |
| Initial slurry volume (mL) | 2000 | 2000 | 2000 |
| Calculated initial slurry mass (g) | 2160 | 2160 | 2160 |
| Calculated initial dry solids (g) | 446.4 | 446.4 | 446.4 |
| Calculated initial water mass (g) | 1713.6 | 1713.6 | 1713.6 |
| Nominal LAC stock-solution volume (mL) | 17.9 | — | — |
| ABTS stock-solution volume (mL) | 12.1 | — | — |
| PAC stock-solution volume (mL) | 42.9 | 42.9 | 42.9 |
| Polymer stock-solution volume (mL) | 40.2 (CMCS) | 40.2 (CMCS) | 35.7 (cPAM) |
| Cumulative collected filtrate at 30 h (mL) | 1702.8 | 1340 | 1392 |
| Central-region cake water content (% dry basis) | 84.94 | 112.19 | 127.23 |

**Note:** Cake water content represents the mean of three subsamples collected from the central region of the same geotextile cake and is not a whole-cake average. Whole-cake wet and dry masses and solids passage through the geotextile were not measured; therefore, the quantities reported in this table do not constitute a closed water-and-solids mass balance.

**Table S2. Two-way ANOVA results forUCS and axial expansion in the laccase-dosage and component-comparison experiments.**

| Experiment | Response variable | Effect | *P* value |
| --- | --- | --- | --- |
| Laccase-dosage | UCS | Treatment | 9.7101 × 10⁻⁹ |
| Laccase-dosage | UCS | Curing age | 8.3313 × 10⁻¹⁵ |
| Laccase-dosage | UCS | Treatment × curing age | 6.7362 × 10⁻⁷ |
| Laccase-dosage | Axial expansion | Treatment | 0.044629 |
| Laccase-dosage | Axial expansion | Curing age | 0.340512 |
| Laccase-dosage | Axial expansion | Treatment × curing age | 0.035069 |
| Component-comparison | UCS | Formulation | 6.5640 × 10⁻¹⁰ |
| Component-comparison | UCS | Curing age | 8.3446 × 10⁻¹⁷ |
| Component-comparison | UCS | Formulation × curing age | 3.3695 × 10⁻⁹ |
| Component-comparison | Axial expansion | Formulation | 2.2691 × 10⁻⁴ |
| Component-comparison | Axial expansion | Curing age | 0.281895 |
| Component-comparison | Axial expansion | Formulation × curing age | 0.014292 |

**Note:** Data were obtained from three independent replicates per treatment (n = 3). The laccase-dosage and sequential-formulation experiments were analyzed separately using two-way ANOVA. Statistical significance was defined at P < 0.05.

**Table S3. Tukey HSD pairwise comparisons among treatments within each solidification experiment and curing age.**

| Experiment | Response variable | Curing age (d) | Comparison | Adjusted P value |
| --- | --- | --- | --- | --- |
| Laccase-dosage | UCS | 3 | OH vs OH+LAC1 | 0.31714 |
|  |  | 3 | OH vs OH+LAC2 | 0.93681 |
|  |  | 3 | OH+LAC1 vs OH+LAC2 | 0.46958 |
|  |  | 7 | OH vs OH+LAC1 | 3.0915 × 10⁻⁴ |
|  |  | 7 | OH vs OH+LAC2 | 0.003209 |
|  |  | 7 | OH+LAC1 vs OH+LAC2 | 0.049464 |
|  |  | 14 | OH vs OH+LAC1 | 5.3901 × 10⁻⁴ |
|  |  | 14 | OH vs OH+LAC2 | 7.4323 × 10⁻⁴ |
|  |  | 14 | OH+LAC1 vs OH+LAC2 | 0.89657 |
| Laccase-dosage | Axial expansion | 3 | OH vs OH+LAC1 | 0.7733 |
|  |  | 3 | OH vs OH+LAC2 | 0.32095 |
|  |  | 3 | OH+LAC1 vs OH+LAC2 | 0.66646 |
|  |  | 7 | OH vs OH+LAC1 | 0.1022 |
|  |  | 7 | OH vs OH+LAC2 | 0.056418 |
|  |  | 7 | OH+LAC1 vs OH+LAC2 | 0.88686 |
|  |  | 14 | OH vs OH+LAC1 | 0.12148 |
|  |  | 14 | OH vs OH+LAC2 | 0.73257 |
|  |  | 14 | OH+LAC1 vs OH+LAC2 | 0.31904 |
| Component-comparison | UCS | 3 | OH vs LAC | 0.99192 |
|  |  | 3 | OH vs LAC+ABTS | 0.99994 |
|  |  | 3 | OH vs LAC+ABTS+CMCS | 0.041024 |
|  |  | 3 | LAC vs LAC+ABTS | 0.98664 |
|  |  | 3 | LAC vs LAC+ABTS+CMCS | 0.027879 |
|  |  | 3 | LAC+ABTS vs LAC+ABTS+CMCS | 0.04415 |
|  |  | 14 | OH vs LAC | 6.8138 × 10⁻⁷ |
|  |  | 14 | OH vs LAC+ABTS | 5.0266 × 10⁻⁵ |
|  |  | 14 | OH vs LAC+ABTS+CMCS | 0.0064716 |
|  |  | 14 | LAC vs LAC+ABTS | 3.7037 × 10⁻⁴ |
|  |  | 14 | LAC vs LAC+ABTS+CMCS | 8.3778 × 10⁻⁶ |
|  |  | 14 | LAC+ABTS vs LAC+ABTS+CMCS | 0.0048929 |
| Component-comparison | Axial expansion | 3 | OH vs LAC | 0.99664 |
|  |  | 3 | OH vs LAC+ABTS | 0.043558 |
|  |  | 3 | OH vs LAC+ABTS+CMCS | 0.22219 |
|  |  | 3 | LAC vs LAC+ABTS | 0.058226 |
|  |  | 3 | LAC vs LAC+ABTS+CMCS | 0.16816 |
|  |  | 3 | LAC+ABTS vs LAC+ABTS+CMCS | 0.002763 |
|  |  | 14 | OH vs LAC | 0.018128 |
|  |  | 14 | OH vs LAC+ABTS | 0.16266 |
|  |  | 14 | OH vs LAC+ABTS+CMCS | 0.99144 |
|  |  | 14 | LAC vs LAC+ABTS | 0.4423 |
|  |  | 14 | LAC vs LAC+ABTS+CMCS | 0.012428 |
|  |  | 14 | LAC+ABTS vs LAC+ABTS+CMCS | 0.10966 |

**Note:** Values are Tukey-adjusted *P* values for pairwise comparisons among treatments within the same curing age. Statistical significance was defined at *P* < 0.05. OH+LAC1 and OH+LAC2 correspond to nominal laccase dosages of 20,000 and 40,000 U kg⁻¹ dry solids, respectively.

**Table S4. One-way ANOVA results for bench-scale dewatering indices.**

| Response variable | P value |
| --- | --- |
| Supernatant turbidity | 3.0291 × 10⁻¹⁸ |
| Zeta potential | 1.6522 × 10⁻⁷ |
| Vacuum-filtrate turbidity | 9.2015 × 10⁻¹² |
| Filter-cake water content | 1.8135 × 10⁻⁷ |

**Note:** Data were obtained from three independent replicates per treatment (*n* = 3). Treatment effects were evaluated using one-way ANOVA. Statistical significance was defined at *P* < 0.05.

**Table S5. Tukey HSD pairwise comparisons for bench-scale dewatering indices.**

| **Comparison** | **Supernatant turbidity** | **Zeta potential** | **Vacuum-filtrate turbidity** | **Filter-cake water content** |
| --- | --- | --- | --- | --- |
| D0 vs D1 | 2.55 × 10⁻¹⁵ | 3.15 × 10⁻⁷ | 0.01686 | 0.22166 |
| D0 vs D2 | 3.22 × 10⁻¹⁵ | 5.58 × 10⁻⁷ | 0.00772 | 4.37 × 10⁻⁵ |
| D0 vs D3 | 2.55 × 10⁻¹⁵ | 3.96 × 10⁻⁷ | 1.67 × 10⁻⁷ | 1.67 × 10⁻⁶ |
| D0 vs D4 | 2.55 × 10⁻¹⁵ | 4.70 × 10⁻⁷ | 1.25 × 10⁻⁹ | 2.76 × 10⁻⁶ |
| D0 vs D5 | 2.55 × 10⁻¹⁵ | 1.60 × 10⁻⁶ | 9.86 × 10⁻¹⁰ | 7.72 × 10⁻⁶ |
| D0 vs D6 | 2.55 × 10⁻¹⁵ | 6.51 × 10⁻⁶ | 1.66 × 10⁻⁵ | 1.10 × 10⁻⁶ |
| D1 vs D2 | 1.71 × 10⁻⁵ | 0.99836 | 1.17 × 10⁻⁵ | 0.00275 |
| D1 vs D3 | 6.39 × 10⁻⁵ | 0.99999 | 2.44 × 10⁻⁵ | 5.23 × 10⁻⁵ |
| D1 vs D4 | 1.95 × 10⁻⁷ | 0.99978 | 4.02 × 10⁻⁸ | 9.64 × 10⁻⁵ |
| D1 vs D5 | 1.97 × 10⁻⁷ | 0.7998 | 2.98 × 10⁻⁸ | 0.000338 |
| D1 vs D6 | 9.59 × 10⁻⁷ | 0.237 | 0.01213 | 3.15 × 10⁻⁵ |
| D2 vs D3 | 5.79 × 10⁻⁹ | 0.99991 | 2.69 × 10⁻⁹ | 0.26502 |
| D2 vs D4 | 1.87 × 10⁻¹⁰ | 1 | 5.75 × 10⁻¹¹ | 0.45182 |
| D2 vs D5 | 1.88 × 10⁻¹⁰ | 0.96944 | 4.75 × 10⁻¹¹ | 0.8744 |
| D2 vs D6 | 5.00 × 10⁻¹⁰ | 0.46756 | 8.05 × 10⁻⁸ | 0.1562 |
| D3 vs D4 | 0.00675 | 1 | 0.00137 | 0.99956 |
| D3 vs D5 | 0.00685 | 0.88878 | 0.000804 | 0.88904 |
| D3 vs D6 | 0.08445 | 0.31747 | 0.02759 | 0.99982 |
| D4 vs D5 | 1 | 0.93712 | 0.9999 | 0.98286 |
| D4 vs D6 | 0.79189 | 0.38837 | 4.56 × 10⁻⁶ | 0.98702 |
| D5 vs D6 | 0.796 | 0.91974 | 3.06 × 10⁻⁶ | 0.72599 |

**Note:** Values are Tukey-adjusted P values. Statistical significance was defined at P < 0.05. D0, untreated OH; D1, PAC+CMCS; D2, LAC+PAC+CMCS; D3, LAC+ABTS+PAC+CMCS; D4, PAC+cPAM; D5, LAC+PAC+cPAM; D6, LAC+ABTS+PAC+cPAM.

**Table S6. One-way ANOVA and Tukey HSD results for DOC concentrations.**

| Analysis | Comparison / Effect | P value |
| --- | --- | --- |
| One-way ANOVA | Treatment effect | 1.5139 × 10⁻⁷ |
| Tukey HSD | OH vs OH+LAC | 3.5101 × 10⁻⁶ |
| Tukey HSD | OH vs OH+LAC+ABTS | 1.1996 × 10⁻⁷ |
| Tukey HSD | OH+LAC vs OH+LAC+ABTS | 1.6997 × 10⁻⁵ |

**Note:** Data were obtained from three independent replicates per treatment (n = 3). DOC concentrations were evaluated using one-way ANOVA followed by Tukey’s honestly significant difference (HSD) test. Pairwise P values are Tukey-adjusted. Statistical significance was defined at P < 0.05.
